# Continuous Dynamic Registration of Microvascularization of Liver Tumors with Contrast-Enhanced Ultrasound

**DOI:** 10.1155/2014/347416

**Published:** 2014-06-02

**Authors:** Lukas Philipp Beyer, Benedikt Pregler, Isabel Wiesinger, Christian Stroszczynski, Philipp Wiggermann, Ernst-Michael Jung

**Affiliations:** Department of Radiology, University Medical Center of Regensburg, Franz-Josef-Strauß-Allee 11, 93053 Regensburg, Germany

## Abstract

*Aim.* To evaluate the diagnostic value of quantification of liver tumor microvascularization using contrast-enhanced ultrasound (CEUS) measured continuously from the arterial phase to the late phase (3 minutes). *Material and Methods. *We present a retrospective analysis of 20 patients with malignant (*n* = 13) or benign (*n* = 7) liver tumors. The tumors had histopathologically been proven or clearly identified using contrast-enhanced reference imaging with either 1.5 T MRI (liver specific contrast medium) or triphase CT and follow-up. CEUS was performed using a multifrequency transducer (1–5 MHz) and a bolus injection of 2.4 mL sulphur hexafluoride microbubbles. A retrospective perfusion analysis was performed to determine TTP (time-to-peak), RBV (regional blood volume), RBF (regional blood flow), and Peak. * Results.* Statistics revealed a significant difference (*P* < 0.05) between benign and malignant tumors in the RBV, RBF, and Peak but not in TTP (*P* = 0.07). Receiver operating curves (ROC) were generated for RBV, RBF, Peak, and TTP with estimated ROC areas of 0.97, 0.96, 0.98, and 0.76, respectively. * Conclusion.* RBV, RBF, and Peak continuously measured over a determined time period of 3 minutes could be of valuable support in differentiating malignant from benign liver tumors.

## 1. Introduction

The radiomorphological differentiation between benign and malignant liver tumors with contrast-enhanced ultrasound is based on a continuous, dynamic evaluation of microvascularization of the tumor [1] and has been shown to be extremely reliable [2, 3].

Malignant liver tumors (HCC, CCC, and metastasis) often have an irregular, sometimes chaotic, early-arterial and arterial (15–45 secs) microvascularization showing a hyperenhancement or rim enhancement [4] in the arterial phase and a hypoechogenic pattern in the portal-venous and the late (2–5 mins) venous phases [4–6]. Metastasis can be hypovascular (e.g., adenocarcinoma) with necrotic, nonenhanced areas or hypervascular (e.g., neuroendocrine tumors) with complete or partial hyperenhancement in the arterial phase. A common feature of almost all metastases is hypo- or nonenhancement in the portal and late phases [7–9].

Most benign hepatic lesions (haemangioma, focal nodular hyperplasia, and adenoma) show a rapid enhancement in the arterial phase with a prolonged enhancement in the late venous phase. They display mainly not only hyperechogenic but also isoechogenic patterns in the late phase [4–6]. Classical patterns include nodular, wheel-spike, or mixed accumulations as seen in haemangioma, focal nodular hyperplasia (FNH), and adenoma, respectively [5].

The use of the 2nd generation contrast medium, for example, sulphur hexafluoride microbubbles (SonoVue, Bracco, Italy), enables the detection of strictly intravascular, capillary microvascularization from the early-arterial phase to the late phase [10]. Continuously recorded images can be used for a computer-assisted perfusion analysis.

Histopathologic findings obtained from percutaneous biopsy or surgical specimens are recommended as the gold standard in the characterization of malignant liver tumors. The imaging techniques contrast-enhanced CT (ceCT) and contrast-enhanced MRI (ceMRI) have been proven valuable in the characterization of benign liver tumors. The use of liver specific contrast medium is preferable, and long-term radiographic follow-up is recommended to confirm the stability of the lesion.

Recommendations for the characterization of hepatocellular carcinoma (HCC) are different and depend on the size of the lesion [11, 12]. These have been issued in a guideline from the World Federation for Ultrasound in Medicine and Biology and the European Federation of Societies for Ultrasound in Medicine and Biology [11] based on the DEGUM (Deutsche Gesellschaft für Ultraschall in der Medizin) multicenter study findings, which emphasizes the value of CEUS in the diagnostic of focal liver lesions [2].

Therefore, the aim of this pilot study was to, for the first time, quantify the perfusion of histopathologically proven or radiomorphologically (ceCT/ceMRI) clearly identified liver tumors over a determined time period of 3 minutes and to evaluate the diagnostic value of the calculated perfusion parameters.

## 2. Materials and Methods

This study comprised 20 patients, of whom 13 had malignant hepatic lesions and 7 had benign hepatic lesions. The mean age of the patients with malignancy was 62.39 (SD 7.54) years, and for benignity it was 47.96 (SD 14.49) years. Eight patients were females (Table 1).

After undergoing CEUS, the malignancy was confirmed by histopathology in 5 cases and by clinicoradiological follow-up in 8 cases: ceMRI in 1 case, ceCT in 3 cases, and a combination of ceCT and ceMRI in the remaining 4 cases. Using ceMRI, benign lesions were confirmed in 6 out of 7 cases, while ceCT was used in the last case (adenoma). Imaging follow-up was performed for a minimum period of 6 months. In addition, all images were separately reviewed by an independent experienced radiologist to confirm the diagnosis of benign/malignant lesions.

Before the procedures, the patient was adequately informed about MRI and CT as well as CEUS, and his/her consent was granted in writing. There were no contrast media intolerances. Approval for this study was obtained from the local ethical board.

Patients with impaired renal function, preexisting strong allergic reactions, and decompensated cardiac failure as well as contraindications for the administration of contrast medium were not included in the study.

### 2.1. US/CEUS

At first, all patients underwent detailed B-scan ultrasound (US). The sonographic examination was performed by an experienced examiner (more than 10.000 abdominal sonographic examinations were done) using a high-end ultrasound machine (LOGIQ E9, GE) and a multifrequency transducer (1–5 MHz, GE Healthcare, Chalfont St. Giles, UK). The whole liver parenchyma was scanned in digital cine sequences using the sweep technique in a transversal and sagittal plane. Color-coded Doppler sonography (CCDS) was used for the evaluation of the unenhanced tumor vascularization. The parameters were optimized for low flow; adapted pulse repetition frequency was selected, less than 1500 Hz. A wall filter was used to reduce motion artifacts.

Following the B-mode and CCDS evaluation of the main tumor lesion, dynamic contrast-enhanced ultrasound examination was carried out with Phase Inversion Technique or Amplitude Modulation in true detection mode, as well as parallel imaging of B-scan and CEUS during the arterial, portal-venous, and late phases for up to 3 minutes.

As ultrasonic second generation contrast agents, sulphur hexafluoride microbubbles (SonoVue, Bracco, Germany) were used. An intravenous bolus of 2.4 mL SonoVue was applied followed by a bolus of 10 mL saline injected through a 20–18 G peripheral cubital cannula. Injection was done by the same assistant in all cases. Digital sequences were recorded as DICOM and afterwards transformed to Audio Video Interleaving (AVI) format.

Retrospectively, one independent examiner used the quantification software QONTRAST (Bracco, Milan, Italy) to obtain contrast-enhanced sonographic perfusion maps for each lesion. The examiner was blinded for the corresponding imaging by CT, MRI, and histology of tumors. QONTRAST is dedicated perfusion quantification software designed for the evaluation of tissue perfusion obtained using contrast-enhanced ultrasound examination in real time. Therefore, an analysis of tissue perfusion based on signal video intensity changes over the time was performed. The software calculates time-intensity curves on a pixel-by-pixel basis. Calculated curves are then fitted to parametric curves and, as a result, parametric maps are obtained. Parametric maps are easy-to-interpret color images describing different aspects of the organ perfusion.

The parameters described in this study are the peak of the signal intensity (Peak), the time-to-peak (TTP) which represents the time of arrival of the contrast agent to its maximum, the regional blood volume (RBV) which is proportional to the area under the curve, and the additional calculation of the regional blood flow (RBF).

## 3. Statistics

Besides descriptive statistics, the nonparametric Mann-Whitney test to compare perfusion parameters was used. The level of significance was *P* < 0.05. For each perfusion measurement, we estimated the area under the empirical receiver operating characteristic curve (AUC) to evaluate the accuracy of separating malignant lesions from benign lesions. 95% confidence intervals are computed for each AUC with Delong's method [13]. For data analysis, the R programming language (R version 3.0.1) and the pROC package [14] were used.

## 4. Results

A dual mode examination (real-time B-mode and CEUS) of the echogenicity and microvascularization was continuously recorded for 3 minutes in all 20 cases. The entire volumes of the tumors were captured, and there were no artifacts. It was not necessary to repeat the injection of contrast media. The evaluation of the echogenicity in B-mode did not reveal any clear patterns for the identification of the tumors. Likewise, the color-coded Doppler flow measurement, adapted for low flow, did not show a specific pattern of vascularization for a definite diagnosis.

Only the use of CEUS enabled the discrimination between malignant and benign tumors. All benign lesions (7 out of 20) showed a continuous contrast enhancement until the late phase. This enhancement appeared nodular for haemangioma and revealed vessels radiating from the central artery to the periphery for FNH. The adenoma showed an enhancement from the tumor border to the center.

All malignant lesions (13 out of 20) showed washout beginning in the portal-venous phase and increasing through the late phase. Metastases showed a circular rim enhancement in the arterial phase. Hepatocellular carcinoma had a nonhomogeneous, sometimes chaotic, pattern of vascularization, and cholangiocellular carcinoma showed a nonhomogeneous parenchyma.

The tumor size ranged from 28 to 111 mm (mean 59 mm, SD 23) for malignant lesions and from 14 to 41 mm (mean 30 mm, SD 9) for benign lesions. Three of the 13 malignant lesions and 4 of the 7 benign lesions were located in the left lobe. For detailed information about the size of the lesions and their perfusion, see Table 2.

The analysis of dynamic microvascularization showed significant differences between malignant and benign lesions regarding RBV, RBF, and Peak (Table 3). Benign lesions showed significantly higher signal intensity (Peak), a higher blood volume (RBV), and a stronger blood flow (RBF). Regarding TTP (time-to-peak), there was a high range with a tendency towards higher TTP for malignant lesions but with no significant differences between benign and malignant lesions.

Receiver operating curves (ROC) were generated for RBV, RBF, Peak, and TTP. The nonparametric estimated ROC areas were 0.97, 0.96, 0.98, and 0.76, respectively. The best cut-off points were determined by the Youden index. They were 33.34, 37.8, 1910.2, and 48.4 for Peak, TTP, RBV, and RBF, respectively (Table 4).

## 5. Discussion

Hepatic lesions show tumor-specific microvascularization patterns, which can be demonstrated using CEUS [5]. Unlike ceCT and ceMRI, it is possible to see a continuous analysis of microvascularization from the early-arterial phase to the late phase using CEUS as shown in this pilot study. In ceCT and ceMRI, this analysis can only be done in sequences and not continuously.

**Figure 1:**
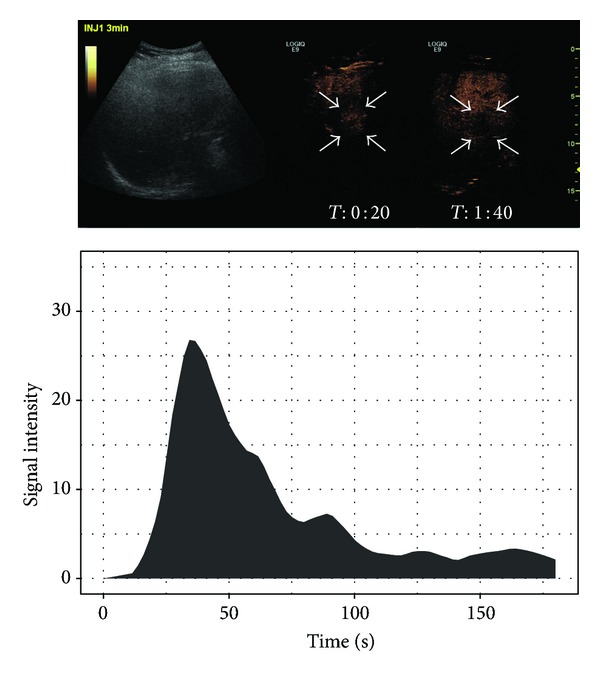
Typical HCC showing a washout starting in the portal-venous phase in B-mode US and CEUS with the corresponding time-intensity curve.

Most malignant tumors show characteristic perfusion patterns with a hyperenhancement (HCC; see Figure 1) or rim enhancement (metastasis, CCC) in the arterial phase [5, 15] and a washout with a non-/hypoenhancement in the portal-venous and late venous phases [5, 16]. The arterial perfusion pattern of HCC is also due to arteriovenous (AV) shunts [17].

Benign lesions can sometimes show a typical pattern of vascularization in the arterial and portal-venous phases. A peripheral-nodular enhancement with subsequent filling can be seen in haemangioma [18], a so-called wheel-spoke pattern in focal nodular hyperplasia and a progressive enhancement from the borders to the center in adenoma [16]. Most benign lesions show no washout with hyper- or less often isoenhancement in the portal-venous and late venous phases [5].

The enhanced microvascularization patterns can not only be visually analyzed and described but also be quantified. A time-intensity curve (TIC) is essential for the quantification. This was continuously performed during this pilot study and, for the first time, over a fixed period of 3 minutes. Perfusion parameters that describe the course of the curve can be derived from the TIC. These analyses can be performed independently and show a high intra- and interobserver agreement [19]. In this study, we were able to show differences in the perfusion parameters Peak, RBV, and RBF that are helpful in distinguishing benign lesions from malignant lesions, thus being valuable in differential diagnosis.

Peak signal intensity (in dB) is the maximum signal intensity reached during the transit of the contrast media. This was significantly greater in benign lesions than in malignant lesions. This concurs with the study findings of Peix et al. [20], who noted a significantly higher Peak in FNH than in HCC. However, a study in mice found that the peak signal intensity (as well as TTP) highly depends on the contrast medium injection speed [21].

**Figure 2:**
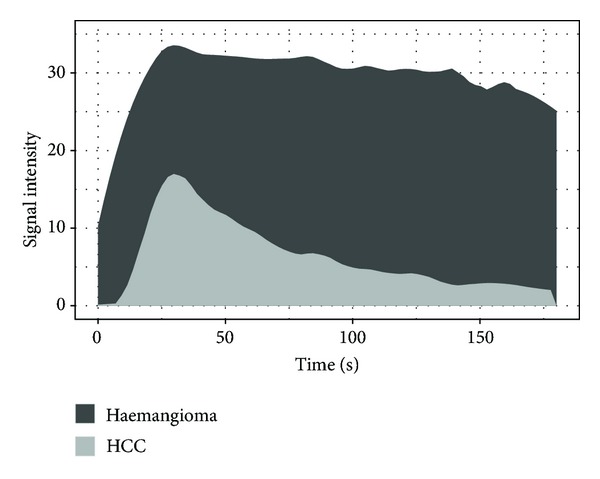
Time-intensity curve of 5 HCCs and 4 haemangiomas after local polynomial regression fitting using LOESS. RBV and RBF are directly proportional to the area under the curve and, therefore, benefit from a continuous registration to the late phase.

The RBV on the other hand was definitely more stable and did not vary with changes in injection speed of contrast media [21]. Benign lesions typically show a steady plateau after reaching the peak in the TIC. This can be explained with their hyperenhancement in the late phase and delayed washout [5, 22]. On the other hand, malignant lesions typically show a hypoenhancement in the late phase; that is, TIC decreases soon after the Peak is attained (see Figure 2). RBV and RBF are directly proportional to the area under the TIC [19]. Therefore, in order to highlight the accuracy of RBV and RBF as diagnostic tools, we believe it is important to capture data until the late phase.

There were different limitations in our present study such as using a heterogeneous collection of different entities of liver tumors or the perfusion software which is not anywhere available. Due to technical limitations, images can only be stored continuously for up to 3 minutes even on high-end ultrasound machines. Further studies with new machines may extend this time frame and further improve the accuracy of perfusion quantification. An experienced examiner and the special technology are fundamental for this ultrasound technique. Another limitation is that the described vascular patterns can only be seen in a part of the lesions depending on the amount of necrosis.

RBV, RBF, and Peak of CEUS in benign and malignant liver tumors, continuously measured over a determined time period of 3 minutes, were significantly different and show high specificity and sensitivity as classifiers. Therefore, quantification of long time perfusion could be valuable in differentiating benign from malignant liver tumors. This opens the field for further dynamic investigations of microvascularization of liver tumors in multicenter studies.

## Figures and Tables

**Table 1:** Characteristics of the study population.

	*n*	Mean age	Min/max age	Female
Benign	7	48	31/75	5
Malignant	13	62	51/72	3
All	20	57	31/75	8

**Table 2:** Size of the lesions (in mm) and their perfusion parameters.

	*n*	Size (SD)	Min/max size	Peak (SD)	TTP (SD)	RBV (SD)	RBF (SD)
Adenoma	1	35	NA	52.6	20.4	4818.5	70.1
CCC	3	65 (22)	50/90	9.4 (5.0)	87.0 (48.8)	920.1 (290.5)	9.9 (4.9)
FNH	2	33 (3)	31/35	51.8 (0.1)	29.3 (11.4)	4660.3 (439.0)	67.3 (2.2)
Haemangioma	4	27 (12)	14/41	45.7 (9.3)	30.3 (6.8)	4426.8 (2050.3)	58.8 (16.2)
HCC	5	66 (26)	46/111	21.5 (12.5)	46.5 (36.5)	1258.0 (576.6)	25.6 (15.4)
Liposarcoma	1	45	NA	3.4	122.1	579.5	3.3
Metastasis	4	50 (25)	28/85	26.5 (14.9)	47.7 (36.8)	2036.9 (1327.8)	33.2 (19.6)

Benign lesions: focal nodular hyperplasia (FNH), adenoma, and haemangioma. Malignant lesions: hepatocellular carcinoma (HCC), cholangiocellular carcinoma (CCC), metastases, and liposarcoma. SD: standard deviation.

**Table 3:** Differences in microvascularization between benign and malignant lesions.

	Benign	Malignant	*P*
Peak (SD)	48.4 (7.4)	18.9 (13.3)	<0.001
TTP (SD)	28.6 (7.6)	62.1 (42.5)	=0.07
RBV (SD)	4549.5 (1469.8)	1367.5 (906.5)	<0.001
RBF (SD)	62.8 (12.6)	22.6 (17.1)	<0.001

**Table 4:** Best cut-off values as determined by the Youden index.

	Threshold	Specificity	Sensitivity
Peak	33.34	1.0	0.92
TTP	37.8	1.0	0.46
RBV	1910.2	1.0	0.85
RBF	48.4	0.86	0.92
